# Increased lesion depth, higher body mass index and older age are risk factors for osteoarthritis during long-term follow-up in patients with osteochondritis dissecans of the knee

**DOI:** 10.1007/s00402-022-04638-4

**Published:** 2022-09-28

**Authors:** Elina Ekman, Sepe Nevalainen, Elina Karjalainen, Ia Kohonen, Jimi Vuohelainen, Tiia Rissanen, Ari Itälä

**Affiliations:** 1 Department of Orthopaedic Surgery, Turku University Hospital, University of Turku, Kiinamyllynkatu 4-8, 20521 Turku, Finland; 2 University of Turku, Turku, Finland; 3 Medical Imaging Centre of Southwest Finland, Turku University Hospital, Turku, Finland; 4 Department of Clinical Medicine, Biostatistics, University of Turku, Turku, Finland; 5 Department of Orthopaedics, Pihlajalinna Hospital, Turku, Finland

**Keywords:** Osteochondritis dissecans, Knee, Cartilage, Osteoarthritis, Treatment outcome, Patient-reported outcome measures

## Abstract

**Introduction:**

To report on the long-term prognosis of osteochondritis dissecans (OCD) patients regarding radiological and patient-reported outcomes and to analyze possible risk factors.

**Materials and methods:**

All patients diagnosed with knee OCD between 2004 and 2014 with radiographic Kellgren–Lawrence (K–L) grades 0–2 at the time of diagnoses, ability to understand the language of the interview, and willingness to participate in the study were retrospectively reviewed. Current knee radiographs and the Knee Injury and Osteoarthritis Outcome Score (KOOS) questionnaire were prospectively collected between May 2020 and March 2021. The extent of osteoarthritis (OA) and KOOS questionnaire results were evaluated.

**Results:**

90 patients (103 knees) with a mean age of 21 years (range 6–60) were included. The mean follow-up time was 12 years (range 7–20). 24 knees (23%) were treated conservatively, and 79 knees (77%) operatively. At the time of diagnoses, 90% of the patients had K–L grades of 0–1; during the follow-up period, 45% of the patients showed radiological progression of OA. Patient body mass index (BMI) (*p* = 0.004; 95% CI 0.25–0.29), age (*p* = 0.003; 95% CI 0.18–0.30), operative treatment (*p* = 0.0075; 95% CI 0.41–0.65) and lesion depth (*p* = 0.0007) were statistically significantly connected to K–L grade change. Patients with no progression in joint space narrowing had statistically significantly better overall KOOS scores (*p* = 0.03; 95% CI 0.77–0.88) than patients whose K–L grades worsened.

**Conclusions:**

During the long-term follow-up of 12 years, patients with knee OCD had good clinical results. Lac of radiological progression of cartilage degeneration was noted in 55% of the patients, regardless of treatment method. Lesion depth, higher BMI and older age were associated with the progression of OA. The progression of OA was related to a worsening of functional scores.

**Level of evidence:**

IV.

## Introduction

Most commonly, OCD is a condition seen in the knee joint; however, other joints might also be affected. The etiology of OCD remains unknown [1, 2]. The most widely accepted etiological hypothesis is that of repetitive microtrauma, although inflammation, ischemia, aseptic avascular necrosis of the subchondral bone, femoro-tibial malalignment, genetic factors, and growth disturbances have also been suggested. Yet, a multifactorial etiology is considered likely [3].

In OCD, a bone fragment and the overlying articular cartilage detach from the subchondral bone. This can result in a loose body and a defect in the articular surface at worst leading to the development of early onset OA [4–6]. Preserving the integrity of the articular cartilage is intended to prohibit this cascade and prevent the development of OA, leading to better functional outcomes [7].

The treatment of OCD varies from conservative to operative depending on the age of the patient and the size, location and stability of the OCD fragment [8]. Juvenile OCD (JOCD) occurs in a young skeletally immature patient with open physes. Adult OCD (AOCD) occurs in skeletally mature adults with closed physes [9]. As compared to AOCD, JOCD has a better prognosis and can often be treated conservatively [6, 10]. Operative treatment is preserved for JOCD not responding to conservative treatment and patients with an unstable OCD. A variety of operative methods to preserve the articular cartilage have been proposed with varying results [4]. Yet, the overall long-term prognosis of OCD of the knee joint, regardless of the treatment method remains unclear.

The primary aim of this study was to report on the long-term prognosis of OCD patients regarding radiological and patient-reported outcomes. The secondary aim was to analyze possible risk factors. We hypothesized that in knee OCD the radiological and patient-reported outcome during long-term follow-up would in general be good but certain patient-related risk factors for compromised outcome could be recognized.

## Materials and methods

**Fig. 1:**
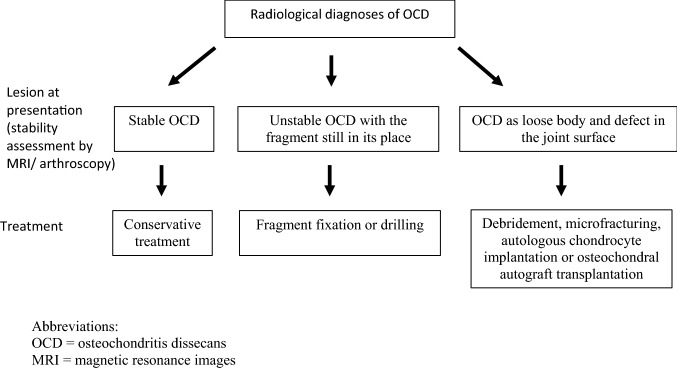
The decision-making process between conservative and operative treatment and the operative method used. *OCD* osteochondritis dissecans, *MRI* magnetic resonance images

A retrospective review of all patients diagnosed with OCD of the knee at Turku University Hospital, Turku, Finland, between January 1st 2004 and December 31st 2014 was conducted. The electronic patient record system (Uranus Miranda, CGI Finland) was searched using the diagnosis code ICD-10: M93.2 to identify both operatively and conservatively treated patients. The inclusion criteria were: knee OCD diagnosed during the study period, radiographic K–L grades 0–2 at the time of diagnoses, ability to understand the language of the interview, and willingness to participate in the study. The information retrieved from the medical charts included patients’ sex, BMI, age at the time of the diagnosis, OCD side, duration of knee symptoms (such as pain and swelling), and treatment method (conservative or operative). For conservative treated patients, BMI was calculated at the time of diagnoses and for operatively treated patients at the time of operation. The decision-making process between conservative and operative treatment and the operative methods used are presented in Fig. 1. Additionally, the knee radiographs and magnetic resonance images (MRI) taken at the time of diagnoses were retrieved from the radiology database for further analyses.

All study patients were called in for current knee radiographs with the knee in a semi-flexed (20°) position (standing PA, lateral and mechanical axis) for the long-term follow-up between May 2020 and March 2021. This imaging technique was also used in the knee radiographs taken at the time of diagnoses making the radiographs comparable. Study patients were also asked to complete the KOOS questionnaire during the same time frame. The KOOS is a self-reported assessment tool consisting of 42 questions distributed among five separately scored subscales: symptoms, pain, activities of daily living (ADL), function in sport and recreation (sport), and knee-related quality of life (QoL). A score ranging from 0 to 100, where 100 represents the best result, is calculated for each subscale [11].

Cartilage changes were estimated based on standardized radiographs by an experienced musculoskeletal radiologist (IK) using the Philips Vue PACS system workstation (Version 11.4; Philips, Rochester, NY, US). For the patients with only MRI taken at the time of diagnoses (*n* = 3), the cartilage changes were estimated based on them. The potential progression of OA was estimated by comparing the diagnostic imaging and current radiographs. The radiologist was not otherwise involved in the patient care or data collection. The extent of OA of the knee was graded according to the Kellgren–Lawrence OA scale. Scores are not compartment-specific; therefore, a patient with a grade-3 change in the patellofemoral compartment who underwent some form of OCD treatment (conservative or operative) on the medial compartment was still recorded as having a grade-3 change. The mechanical alignment of the leg was assessed based on full-length, weight-bearing radiographs within an accuracy of one degree by a musculoskeletal radiologist (IK). Knee MRI studies taken at the time of diagnoses were used to determine, within an accuracy of 1 mm, the size, depth and location of the defect via the same radiologist (IK). The MRI studies were obtained using a 3.0 T scanner in 13 patients, a 1.5 T scanner in 63 patients and low-magnetic-field equipment (1 T or below) in nine patients. For those patients who did not undergo an MRI study (*n* = 14) at the time of diagnoses, radiographs and medical records from their operations (if treated operatively) were used to determine the sizes and location of the defects. The study was approved by the Regional Ethical Review Board in Turku (Dnro ETMK 128/2019).

### Statistical analysis

The associations between the mean KOOS score and the study variables (sex, age, BMI, size of defect, location of defect, operation, depth of defect, OCD in one or two knees, knee alignment axis, K–L grade from the radiographs at the time of follow-up and a variable indicating a change between the K–L grade obtained from the radiographs at the time of diagnoses and the K–L grade obtained from the radiographs at the time of follow-up) were summarized with descriptive statistics and studied one by one with a Spearman correlation and Kruskal–Wallis test (for categorical variables). The associations between a change in the K–L grade (a two-class categorical variable intended to examine the mean difference between patients whose cartilage degeneration had worsened and those whose injuries had remained the same) and explanatory variables (KOOS mean and subscales, gender, age, BMI, size of defect, location of defect, operation, depth of defect, knee alignment axis and K–L grade from the radiographs at the time of follow-up) were studied with a Kruskal–Wallis test and Fisher’s exact test.

The normality of the variables was evaluated visually and tested with the Shapiro–Wilk test. Due to the non-normality of the continuous variables, non-parametric methods were used. The statistical significance level was set at 0.05 in all tests (two-tailed), and 95% confidence intervals (CI) were calculated. The analyses were performed using the SAS system, Version 9.4, for Windows (SAS Institute Inc., Cary, NC, USA).

## Results

Altogether, 90 patients met the inclusion criteria and formed the study group with mean follow-up time of 12 years (range 7–20) from diagnosis. Thirteen (14%) patients had OCD lesions in both knees and were included twice in the data as two separate cases, thus forming a final cohort of 103 knees. Of the study cohort, 24 knees (23%) were treated conservatively and 79 knees (77%) were treated operatively, with a variation of operative methods. At the time of follow-up, current radiographs were taken of 90 knees. Patient and lesion demographics are presented in Table 1.

**Table 1:** Patient and lesion demographics

Patients	*n*	
Gender, female/male, %	37/53	41/59
Age (years), median (range)	90	21 (6–60)
BMI, median (range)	54	24 (15–40)
Knee, right/ left, %	54/49	52/48
Duration of symptoms (months), median (range)	44	8 (0–60)
Alignment, 0 or varus/valgus, %	45/27	63/37
OCD lesions		
Total surface area (cm^2^), median (range)	103	2.5 (1.2–9.9)
Dept (mm), mean (SD)	89	2.1 (1.6)
Location, medial/lateral femoral condyle, %	91/11	89/11

*BMI* body mass index, *OCD* osteochondritis dissecans, *SD* standard deviation

At the time of diagnoses, 90% of patients did not have any significant cartilage degeneration (K–L grades of 0–1); at the time of follow-up, 45% of the knees showed the radiological progression of joint space narrowing (at least one grade level on the K–L scale) (Table 2). No progression of joint space narrowing was noted in 55% of the knees, narrowing by one grade was noted in 29%, by two grades in 14% and by three grades in 2%. No patient had joint space narrowing by four grades. Most patients had no change in their K–L grades, and among the patients who had a change, most had their K–L grades worsened by one a single step. Therefore, we compared patients with no change in their K–L grades and patients with a change in their K–L grades (of one, two or three steps). The progression of joint space narrowing was not dependent on K–L grade at the time of diagnoses (n.s.). Also, patient sex (n.s.), the size of the OCD lesion (n.s.), knee alignment axis (n.s.), and location of the defect (medial or lateral condyle) (n.s.) did not affect the progression of OA, as measured by K–L grade change. However, the depth of the lesion (*p* = 0.0007), patient BMI (*p* = 0.004; 95% CI 0.25–0.29), and age at the time of the diagnosis (*p* = 0.003; 95% CI 0.18–0.30) were statistically significantly connected to K–L grade change; deeper lesions, larger BMI, and older age led to larger changes in K–L grade. Also, conservatively treated patients had statistically significantly less progression in their K–L grade change than operatively treated patients (*p* = 0.0075; 95% CI 0.41–0.65).

**Table 2:** KOOS scores for Kellgren–Lawrence grades (at the time of diagnoses and at the final follow-up) and changes in Kellgren–Lawrence grade

Kellgren–Lawrence grade	At diagnoses% (*n*)	KOOS_tot_%	At the final follow-up% (*n*)	KOOS_tot_%	Δ grade % (n)	KOOS_tot_%
0	77 (77)	83	39 (35)	86	55 (48)	88
1	13 (13)	88	30 (27)	87	29 (26)	84
2	10 (10)	88	24 (22)	84	14 (12)	76
3	0		6 (5)	74	2 (2)	80
4	0		1 (1)		0	

*KOOS* Knee Injury and Osteoarthritis Outcome Score, *KOOS*_*tot*_ KOOS with all subscales included, *Δ grade* Kellgren–Lawrence grade change

The KOOS scores at the final follow-up related to the different K–L grades (at the time of diagnoses and at the final follow-up) and to the grade of joint space narrowing are given in Table 2. The mean functional score for KOOS_total_ was 84 at a mean follow-up of 12 years after OCD treatment. Patients with no progression in joint space narrowing had statistically significantly better overall KOOS scores (*p* = 0.03; 95% CI 0.77–0.88) than patients whose K–L grades worsened. This was evident in KOOS_total_ and the subscales for pain (*p* = 0.04; 95% CI 0.86–0.97), sport (*p* = 0.02; 95% CI 0.60–0.80), and ADL (*p* = 0.03; 0.91–1.00) but not the subscales for symptoms (n.s.) and QoL (n.s.). Patient sex (n.s.), BMI (n.s.), age at the time of diagnosis (n.s.), knee alignment axis (n.s.), OCD lesion size (n.s.), OCD lesion dept (n.s.), and location of the defect (n.s.) had no effect on outcomes, as measured by KOOS. There was also no difference in KOOS scores between conservatively and operatively treated patients (n.s.). Whether patients had OCD lesions in both of their knees or just one had no effect on outcome (KOOS_total_ or progression of radiological cartilage changes).

## Discussion

The main finding of the present study was that 55% of patient diagnosed with knee OCD showed no radiological progression of cartilage degeneration during long-term follow-up. Increased lesion depth, higher BMI, and older age were associated with the progression of OA, as measured with a change in K–L grade. The mean functional score of the KOOS_total_ was 84 at a mean follow-up of 12 years after OCD treatment. The progression of joint space narrowing had a negative correlation with patient-reported outcomes measured by KOOS.

In previous literature, long-term results with over 10 years of follow-up regarding knee function and radiological outcome are sparse [12–16]. The literature is given in Table 3. Studies by Martincic et al. [14] and Ekman et al. [13] both included a small percentage of traumatic osteochondral lesions, which may worsen outcomes. The OA percentages in the studies by Twyman et al. [16], Michael et al. [15], and Bruns et al. [12] are likely to be higher than in the current study because of their substantially longer follow-up. However, our results are in line with the two previously mentioned studies with similar follow-up durations [13, 14].

**Table 3:** The literature about radiological outcome and knee function after OCD treatment

Reference	Treatment method	Number of patients	Follow-up (years)	Progression of osteoarthritis	PROM
Twyman et al. [16]	operative (VM) and conservative	22	33	50%	Good 52% Poor 48%
Michael et al. [15]	operative (VM)	24	28	60%	Good 62% Poor 38%
Bruns et al. [12]	operative (VM)	42	10 and 20	44% and 54%	Fair at 10 years and good at 20 years
Martincic et al. [14]	operative (ACI)	33	10	45%	Improvement to preoperative scores
Ekman et al. [13]	operative (OAT)	64	10–11	50%	Good

*OCD* osteochondritis dissecans, *PROM* patient-reported outcome measure, *VM* various methods, *ACI* autologous chondrocyte implantation, *OAT* osteochondral autograft transplantation

In the present study, older age at the time of OCD treatment predicted the progression of OA. As mentioned above, this is consistent with previous studies reporting less OA in JOCD, regardless of the treatment method [10, 12, 17, 18]. It is hypothesized that the remaining growth potential in juvenile patients with open physes leads to better healing capacity in OCD treatment [10].

Higher BMI was associated with an increased risk of developing OA. There are two previous studies investigating the correlation between BMI and OA in knee OCD patients [7, 19]. These studies report a higher incidence of OA in patients with BMIs greater than 25 kg/m^2^, regardless of whether the patients were treated surgically or conservatively. Our study supports these findings. It has been shown that increased BMI results in greater tiobiofemoral contact and shear pressures, leading to an increased risk of knee OA [20, 21].

There is conflicting evidence regarding the connection between OCD lesion size, the progression of OA, and clinical outcomes [13, 22]. Currently, the treatment strategies are based on lesion stability rather than size [4]. In the present study, we found that lesion size did not predict outcome; however, the depth of the lesion had a significant connection with radiological cartilage degeneration. No impact on patient-reported outcome was noted. To the best of our knowledge, there are no previous studies reporting the effect of lesion depth on OCD outcomes. Theoretically, the depth of the lesion correlates to the area of compromised blood supply for the healing bone on bone interface and might therefore be a prognostic factor for the outcome. However, this theory remains open for further study. Selection bias may explain the finding of less OA in conservatively treated patients in that JOCD and stable lesions are likely to be treated conservatively and have good prognoses [10].

There was a significant correlation between the progression of joint space narrowing and patient-reported outcome results. Patients with joint space narrowing had worse functional outcomes, as measured by KOOS, than patients with no joint space narrowing. Based on our results, it seems that even mild/moderate radiological progression is related to the worsening of functional results. One previous study of knee OCD with long-term follow-up reported worse functional outcomes in patients with joint space narrowing [13]. Other similar studies did not investigate the correlation between functional results and the potential development of OA [12, 14–16]. However, functional results were reported to be good in over 50% of patients in two studies [15, 16]. Two other studies reported functional results improving over time, even though the incidence of OA also increased over time [12, 14]. In the previous literature, the correlation between knee symptoms and radiological knee OA in general is unclear [23–27], and patient-related factors, such as pain sensitivity and central sensitization, have been suggested to be connected to this phenomenon [28].

This is a single-center retrospective study. The results describe the long-term patient-reported outcomes and radiological results of knee OCD treatment. We also aimed to determine risk factures for compromised outcome. Our aim was not to compare the results of different treatment methods or provide recommendations regarding how knee OCDs should be treated. The study has several limitations. Our data were collected in a retrospective manner; thus, the initial patient-reported outcome measures were not available for the comparison of functional outcomes. Also, three patients had only MRI studies taken at the time of diagnoses making the comparison to radiographs regarding OA uncertain. However, this comprises only 3% of the study cohort and therefore we consider the risk for significant bias to be small. Additionally, there are missing data as often is the case in retrospective studies. The analysis was carried out with the data available. Also, no differentiation between surgical procedures was made; patients were treated as current treatment recommendations advised and assessed as one group. However, our study provides surgeons and patients with OCD treatment prognoses in the long run and also knowledge of risk factors regarding treatment outcome. In the future, the results of current treatment concepts, that is conservative, palliative, reparative and restorative treatment, will require further study.

## Conclusions

During a long-term follow-up of 12 years, patients with knee OCD had good clinical results. Lac of radiological progression of cartilage degeneration was noted in 55% of the patients, regardless of treatment method. Lesion depth, higher BMI and older age were associated with the progression of OA. The progression of OA was related to a worsening of functional scores.

## References

[1] Federico DJ, Lynch JK, Jokl P (1990). Osteochondritis dissecans of the knee: a historical review of etiology and treatment. Arthroscopy.

[2] Polousky JD (2011). Juvenile osteochondritis dissecans. Sports Med Arthrosc Rev.

[3] Chau MM, Klimstra MA, Wise KL, Ellermann JM, Toth F, Carlson CS (2021). Osteochondritis dissecans: current understanding of epidemiology, etiology, management, and outcomes. J Bone Joint Surg Am.

[4] Bruns J, Werner M, Habermann C (2018). Osteochondritis dissecans: etiology, pathology, and imaging with a special focus on the knee joint. Cartilage.

[5] Buckwalter JA, Anderson DD, Brown TD, Tochigi Y, Martin JA (2013). The roles of mechanical stresses in the pathogenesis of osteoarthritis: implications for treatment of joint injuries. Cartilage.

[6] Linden B (1977). Osteochondritis dissecans of the femoral condyles: a long-term follow-up study. J Bone Joint Surg Am.

[7] Sanders TL, Pareek A, Obey MR, Johnson NR, Carey JL, Stuart MJ (2017). High rate of osteoarthritis after osteochondritis dissecans fragment excision compared with surgical restoration at a mean 16-year follow-up. Am J Sports Med.

[8] Jones MH, Williams AM (2016). Osteochondritis dissecans of the knee: a practical guide for surgeons. Bone Joint J.

[9] Cahill BR (1995). Osteochondritis dissecans of the knee: treatment of juvenile and adult forms. J Am Acad Orthop Surg.

[10] Hefti F, Beguiristain J, Krauspe R, Moller-Madsen B, Riccio V, Tschauner C (1999). Osteochondritis dissecans: a multicenter study of the European Pediatric Orthopedic Society. J Pediatr Orthop B.

[11] Roos EM, Toksvig-Larsen S (2003). Knee injury and Osteoarthritis Outcome Score (KOOS)—validation and comparison to the WOMAC in total knee replacement. Health Qual Life Outcomes.

[12] Bruns J, Rayf M, Steinhagen J (2008). Longitudinal long-term results of surgical treatment in patients with osteochondritis dissecans of the femoral condyles. Knee Surg Sports Traumatol Arthrosc.

[13] Ekman E, Makela K, Kohonen I, Hiltunen A, Itala A (2018). Favourable long-term functional and radiographical outcome after osteoautograft transplantation surgery of the knee: a minimum 10-year follow-up. Knee Surg Sports Traumatol Arthrosc.

[14] Martincic D, Radosavljevic D, Drobnic M (2014). Ten-year clinical and radiographic outcomes after autologous chondrocyte implantation of femoral condyles. Knee Surg Sports Traumatol Arthrosc.

[15] Michael JW, Wurth A, Eysel P, Konig DP (2008). Long-term results after operative treatment of osteochondritis dissecans of the knee joint-30 year results. Int Orthop.

[16] Twyman RS, Desai K, Aichroth PM (1991). Osteochondritis dissecans of the knee. A long-term study. J Bone Joint Surg Br..

[17] Cahill BR, Phillips MR, Navarro R (1989). The results of conservative management of juvenile osteochondritis dissecans using joint scintigraphy. A prospective study. Am J Sports Med.

[18] Trinh TQ, Harris JD, Flanigan DC (2012). Surgical management of juvenile osteochondritis dissecans of the knee. Knee Surg Sports Traumatol Arthrosc.

[19] Sanders TL, Pareek A, Johnson NR, Carey JL, Maak TG, Stuart MJ (2017). Nonoperative management of osteochondritis dissecans of the knee: progression to osteoarthritis and arthroplasty at mean 13-year follow-up. Orthop J Sports Med.

[20] Harding GT, Dunbar MJ, Hubley-Kozey CL, Stanish WD, Astephen Wilson JL (2016). Obesity is associated with higher absolute tibiofemoral contact and muscle forces during gait with and without knee osteoarthritis. Clin Biomech (Bristol, Avon).

[21] Reyes C, Leyland KM, Peat G, Cooper C, Arden NK, Prieto-Alhambra D (2016). Association between overweight and obesity and risk of clinically diagnosed knee, hip, and hand osteoarthritis: a population-based cohort study. Arthritis Rheumatol.

[22] Gudas R, Kalesinskas RJ, Kimtys V, Stankevicius E, Toliusis V, Bernotavicius G (2005). A prospective randomized clinical study of mosaic osteochondral autologous transplantation versus microfracture for the treatment of osteochondral defects in the knee joint in young athletes. Arthroscopy.

[23] Bedson J, Croft PR (2008). The discordance between clinical and radiographic knee osteoarthritis: a systematic search and summary of the literature. BMC Musculoskelet Disord.

[24] Hannan MT, Felson DT, Pincus T (2000). Analysis of the discordance between radiographic changes and knee pain in osteoarthritis of the knee. J Rheumatol.

[25] Lawrence JS, Bremner JM, Bier F (1966). Osteo-arthrosis. Prevalence in the population and relationship between symptoms and X-ray changes. Ann Rheum Dis.

[26] Laxafoss E, Jacobsen S, Gosvig KK, Sonne-Holm S (2010). Case definitions of knee osteoarthritis in 4,151 unselected subjects: relevance for epidemiological studies: the Copenhagen Osteoarthritis Study. Skelet Radiol.

[27] Neogi T, Felson D, Niu J, Nevitt M, Lewis CE, Aliabadi P (2009). Association between radiographic features of knee osteoarthritis and pain: results from two cohort studies. BMJ.

[28] Finan PH, Buenaver LF, Bounds SC, Hussain S, Park RJ, Haque UJ (2013). Discordance between pain and radiographic severity in knee osteoarthritis: findings from quantitative sensory testing of central sensitization. Arthritis Rheum.

